# Candidate gene family-based and case-control studies of susceptibility to high *Schistosoma mansoni* worm burden in African children: a protocol

**DOI:** 10.12688/aasopenres.13203.2

**Published:** 2021-12-15

**Authors:** Oscar A. Nyangiri, Sokouri A. Edwige, Mathurin Koffi, Estelle Mewamba, Gustave Simo, Joyce Namulondo, Julius Mulindwa, Jacent Nassuuna, Alison Elliott, Kévin Karume, Dieudonne Mumba, P.L.A.M Corstjens, M. Casacuberta-Partal, G.J. van Dam, Bruno Bucheton, Harry Noyes, Enock Matovu, TrypanoGEN+ Research Group of the H3Africa Consortium

**Affiliations:** 1 College of Veterinary Medicine, Animal Resources and Biosecurity, Makerere University, Kampala, Uganda; 2 Université Jean Lorougnon Guédé (UJLoG) de Daloa, Daloa, Cote d'Ivoire; 3 Faculty of Science, University of Dschang, Dschang, Cameroon; 4 Medical Research Council/Uganda Virus Research Institute and London School of Hygiene & Tropical Medicine Uganda Research Unit, Entebbe, Uganda; 5 London School of Hygiene and Tropical Medicine, London, WC1E, UK; 6 Institut National de Recherche Biomedicale, Kinshasa, Democratic Republic of the Congo; 7 Dept. of Cell and Chemical Biology, Leiden University Medical Center, Leiden, The Netherlands; 8 Dept. of Parasitology, Leiden University Medical Center, Leiden, The Netherlands; 9 Institut de Recherche pour le Développement (IRD), IRD-CIRAD, Montpellier, France; 10 Centre for Genomic Research, University of Liverpool, Liverpool, UK

**Keywords:** S. mansoni, infection intensity, family based linkage, haplotypes, candidate genes

## Abstract

**Background: **Approximately 25% of the risk of *Schistosoma mansoni* is associated with host genetic variation. We will test 24 candidate genes, mainly in the T _h_2 and T _h_17 pathways, for association with *S. mansoni* infection intensity in four African countries, using family based and case-control approaches.

**Methods: **Children aged 5-15 years will be recruited in *S. mansoni* endemic areas of Ivory Coast, Cameroon, Uganda and the Democratic Republic of Congo (DRC). We will use family based (study 1) and case-control (study 2) designs. Study 1 will take place in Ivory Coast, Cameroon, Uganda and the DRC. We aim to recruit 100 high worm burden families from each country except Uganda, where a previous study recruited at least 40 families. For phenotyping, cases will be defined as the 20% of children in each community with heaviest worm burdens as measured by the circulating cathodic antigen (CCA) assay. Study 2 will take place in Uganda. We will recruit 500 children in a highly endemic community. For phenotyping, cases will be defined as the 20% of children with heaviest worm burdens as measured by the CAA assay, while controls will be the 20% of infected children with the lightest worm burdens. Deoxyribonucleic acid (DNA) will be genotyped on the Illumina H3Africa SNP (single nucleotide polymorphisms) chip and genotypes will be converted to sets of haplotypes that span the gene region for analysis. We have selected 24 genes for genotyping that are mainly in the Th2 and Th17 pathways and that have variants that have been demonstrated to be or could be associated with *Schistosoma* infection intensity.

**Analysis:** In the family-based design, we will identify SNP haplotypes disproportionately transmitted to children with high worm burden. Case-control analysis will detect overrepresentation of haplotypes in extreme phenotypes with correction for relatedness by using whole genome principal components.

## Abbreviations

BMI: Body mass index

CAA: Circulating anodic antigen

CCA: Circulating cathodic antigen

DRC: Democratic Republic of Congo

FBAT: Family based association test

H3Africa: Human Heredity and Health in Africa research consortium

KK: Kato Katz

LaVIISWA: Lake Victoria Island intervention study on worms and allergy

LOD: Logarithm of odds

MAF: Minor allele frequency

MDA: Mass drug administration

MUAC: Mid Upper arm circumference

PCR: Polymerase chain reaction

POC-CCA: Point of care-circulating cathodic antigen test

PZQ: Praziquantel

QTL : Quantitative trait locus/loci

RDT: Rapid detection test

REC: Research ethics committee

SM1: Schistosoma mansoni susceptibility locus 1

SM2: Schistosoma mansoni susceptibility locus 2

SRR: Sibling Recurrence Risk

TDT: Transmission disequilibrium test

Th1: T helper cell 1 cells/pathway of immunity

Th2: T helper cell 2 cells/pathway of immunity

Th17: T helper 17 cells/pathway of immunity

UCP-LF: Upconverting phosphor lateral flow

UNCST: Uganda National Council of Science and Technology

UVRI: Uganda Virus Research Institute

WHO: World Health Organization

## Introduction

Schistosomiasis is a major parasitic disease with prevalence estimates ranging from about 140 million to more than 250 million people worldwide, while nearly one billion are at risk ( Colley *et al*., 2014; LoVerde, 2019; McManus *et al*., 2018). In 2016, at least 206.5 million people required preventive treatment and more than 40 million people were treated for the parasite according to the WHO. Currently, 90% of all cases, and most severe cases, are in sub-Saharan Africa ( Beltrame *et al*., 2017; Mayaka, 2001). There are 8 to 10 million new cases reported each year worldwide, with 800,000 deaths per year globally and between 200,000 and 400,000 deaths per year in the sub-Saharan African region ( Saotoing *et al*., 2011; WHO, 2013).

Schistosomiasis is linked to poverty, affects the socio-economic development of populations ( Adenowo *et al*., 2015) and represents a significant burden for developing countries, particularly African countries ( Mnkugwe *et al*., 2020). The major species prevalent in African countries are *S. mansoni*, which causes intestinal schistosomiasis and *S. haematobium*, which causes urinary schistosomiasis ( Colley *et al*., 2014). School age children have the highest worm burdens ( Mnkugwe *et al*., 2020; Saotoing *et al*., 2011).

Mass drug administration (MDA) in school children, snail control programs and improved sanitation are all important to the control of schistosomiasis, but each approach is held back by serious technical difficulties and inadequate resources.

MDA has been the major strategy employed and all children in high prevalence areas are treated irrespective of whether they are infected or not. The frequency of MDA (every six months or every one or two years) is dependent on the prevalence of schistosomiasis in a region ( Montresor & World Health Organization, 2011). MDA could be targeted to the most susceptible children; however, there is an incomplete understanding of host genetics of susceptibility, requiring more studies to guide this strategy.

We have reviewed genetic studies of the human response to schistosome infection and found the associations have been reported between schistosome worm burdens and 24 single nucleotide polymorphisms (SNP) in 11 candidate genes ( Mewamba *et al*., 2021a). Mapping studies have shown that there are at least three quantitative trait loci (QTL) regions of the genome that regulate the response to infection, however the genes that are responsible for these observations have not been identified ( Mewamba *et al*., 2021a). Although candidate gene studies are a powerful strategy for discovering associations between SNP and phenotype, the majority of such observations have not been replicated when studies have been repeated in different populations ( Hirschhorn *et al*., 2002). Whilst two of the 24 SNP associated with schistosomiasis have replicated in additional populations, four have failed to replicate when tested and no attempt has been made to replicate the remaining 18 ( Mewamba *et al*., 2021a). We will undertake replication studies for all eleven of these genes in populations from Ivory Coast, Cameroon, Uganda and the Democratic Republic of Congo that are endemic for *S. mansoni* schistosomiasis and that have not been previously included in association studies ( Table 1). We will test additional genes that are in QTL and that are known to be involved in the response to schistosome infection but have not been previously included in genetic association studies. We will also test genes in the Th _17_ pathway that have not been included in genetic studies of schistosomiasis. We will genotype using the H3Africa Illumina Omni SNP chip that contains approximately 2.5m SNP. This will enable us to construct haplotypes across whole gene regions for association tests, rather than relying on individual SNP. 

**Table 1.** Genes that will be tested for association with worm burden or egg count. Genes that have been previously associated with schistosomiasis are indicated by references to the relevant publications and those that have not previously been tested in a candidate gene study are marked as Novel. Novel genes are annotated with the number of SNP within 5kb of gene that have had a -log p value > 6 for association with phenotype in any GWAS study, data from GWAS central (27/1/2021) ( Beck *et al*., 2020). A brief justification for their inclusion is shown, a fuller justification can be found in Supplementary Tables and in our review of the genetics of human schistosomiasis ( Mewamba *et al*., 2021a). Other genes that have been considered but are not currently included are *CRP(6), IL9(1), CD14(2), CXCL14 (0), IL3 (0), VEGFA(12), CTGF (0), IL22RA2 (0), NOS3 (4), SHH(1)*. The numbers in brackets show the number of SNP with associations in GWAS as in the Novel column in the Table. Amino acid changes functional effects of selected SNP in these genes are in Supplementary_tableVEP1.xls ( Noyes, 2021b).

Gene names	Reference (Novel)	Justifications
*IFNG*	( Gatlin *et al*., 2009)	SM2 Chr6 Region
*IL10*	( Adedoja *et al*., 2018; Grant *et al*., 2011)	
*IL13*	( Gatlin *et al*., 2009)( Grant *et al*., 2012; Isnard *et al*., 2011; Kouriba *et al*., 2005)	SM1 Chr5 Region
*IL4*	( Adedokun *et al*., 2018) ( Gatlin *et al*., 2009)	SM1 Chr5 Region
*IL5*	( Ellis *et al*., 2007)	SM1 Chr5 Region
*STAT6*	( Adedokun *et al*., 2018; He *et al*., 2008)	
*CTLA4*	( Idris *et al*., 2012)	
*FCN2*	( Ouf *et al*., 2012)	
*COLEC11*	( Antony *et al*., 2015)	
*ABO*	( Tiongco *et al*., 2018)	
*IL12B*	Novel (2, Psoriasis)	SM1 chr 5 QTL, Th1 and Th17 pathways
*IL17A*	Novel (0)	Chr 6 6p21-q21 QTL, implicated in granuloma formation
*IL17B*	Novel (0)	SM1 chr 5 QTL, implicated in granuloma formation
*IL25 (IL17E)*	Novel (1)	Implicated in granuloma formation; Th17 pathway
*IL17F*	Novel (1)	Chr 6 6p21-q21 QTL, implicated in granuloma formation
*IL17RA*	Novel (5)	Implicated in granuloma formation; Th17 pathway
*IL1A*	Novel (1)	May regulate Th17 Cell differentiation
*IL1B*	Novel (6)	May regulate Th17 Cell differentiation
*TGFB1*	Novel (11)	Regulates Th17 Cell differentiation
*IL6R*	Novel (20, Asthma, CRP, Fibrinogen)	Chr 1 1p21-q23 QTL Granuloma formation and Th17 pathway
*IL6*	Novel (9)	May also mediate the Th17 Cell amplification
*IL21*	Novel (1, Coeliac)	Mediate the Th17 Cell amplification
*IL23A*	Novel (1, Coeliac)	Stabilize Th17 Cell production
*RNASE3 (ECP)*	( Eriksson *et al*., 2007)	

The study will sample intensively within a small number of communities in four countries (Ivory Coast, Cameroon, Uganda and the Democratic Republic of Congo) where schistosomiasis prevalence is high. Under these conditions case control studies within communities are at risk of being confounded by cryptic relatedness which can cause type 1 errors (false positives). The large number of candidate gene case control studies that have not been replicated may be partially a consequence of population structure ( Mewamba *et al*., 2021a). We will use two strategies to control for population structure: 1) family-based study designs, which are immune to errors caused by population sub-structure ( Laird & Lange, 2009); 2) a case-control design with correction for population structure using principal components calculated from genome wide SNP data.

Estimating intensity of infection is also a problem for schistosomiasis studies. The Kato-Katz method of counting eggs in stool samples is known to lack sensitivity and this is compounded by day to day variation in egg excretions and uneven distribution of eggs across an individual stool ( Kongs *et al*., 2001).

The proposed study aims to improve on previous studies by using:

1) A more reproducible phenotyping method (CCA or CAA)
2) A more systematic selection of candidate genes ( Table 1)
3) A study design that is less sensitive to population structure

The specific study aims are as follows:

1) Test candidate genes listed in Table 1 for association with intensity of *S. mansoni* infection
2) Test if previously published associations are replicated in other populations

The hypotheses we will test are:

i) Variation in Th2 and Th17 pathway genes contribute to variation in *S. mansoni* worm burden
ii) Genes in the Th17 pathway contribute to the association with schistosomiasis at three QTL
iii) Associations found with genes in the SM1 and SM2 QTL are replicable across multiple populations

## Protocol

### Ethical approval and consent to participate

The study will be carried out in Cameroon, Uganda, DRC and Ivory Coast. In Cameroon the study was approved by the National Ethics Committee for research on human health of the Ministry of Public Health of Cameroon with the reference number N ^o^2019/02/1144/CE/CNERSH/SP. The review board of the Molecular Parasitology and Entomology Subunit of the Department of Biochemistry of the Faculty of Science of the University of Dschang gave its approval.

In Uganda, the approval was from the Uganda National Council for Science and Technology (UNCST) number: HS 1183 and the Uganda Virus Research Institute Research Ethics Committee: approval of amendment to perform genetic study on human susceptibility to schistosomiasis Ref: GC/127/18/09/317

In DRC, the ethics review number is COMETH/TKK/PRES/004/2018 approved by the Clinic Ngaliema Ethical Committee of the Health Ministry.

In Ivory Coast, the study was approved by the National Ethics Committee for Life Sciences and Health (cnesvs); approval number N/Ref: 040-19/MSHP/CNESVS-kp. In all countries, since schools are involved, we will consult the ministry of education in conducting the study.

### Study design

Two study designs will be employed in different countries, described in detail below.

Study 1: Family based linkage study; to be conducted in the Democratic Republic of Congo, Ivory Coast, Cameroon and the Uganda Lake Victoria islands.

Study 2: Case control study; to be conducted in the Lake Albert Region of Uganda.

***Study 1: Family based linkage and association study***

***Study sites***

Uganda: previous collections by the Lake Victoria Island Intervention Study on Worms and Allergy (LaVIISWA), conducted on the Lake Victoria islands of Koome.

Ivory Coast: Blolquin and Guiglo (West)

Cameroon: Makenene and Adamawa regions

DRC: Kongo Central (Kisantu and Kimpese)

***Participant recruitment***

Consent will be obtained in two stages.

1) Written collective consent for screening for schistosome worm burden will be provided by school directors or health authorities.
2) Written informed consent for children who meet inclusion criteria to participate in the study will be requested from parents or guardians in the home after children have been screened for intensity of infection at school.

The informed consent forms and case report forms are provided as part of extended data in Harvard Dataverse ( Noyes, 2021a).

Community entry procedures differ in each country as mandated by the respective ethical review committees. In DRC administrative authorities, school inspectors, school directors and teachers were involved, whereas in Ivory Coast administrative authorities, Regional Health Director, Departmental Health Director school inspectors, school directors and teachers were involved. In Cameroon, the following were notified about the study; Ministry of Health and Ministry for Schools for Region, school inspectors, school directors and teachers in participating schools. In Uganda, the ministry of health and village health teams were involved in community entry.

Subsequently collective consent will be obtained from district health authorities or school directors and the date of the screening will be planned. The parents or guardians of children at schools will be informed by school directors of schistosomiasis screening at the school on a given date and invited to let their children participate. In Cameroon, DRC and Ivory Coast, schools were chosen that were in high *S. mansoni* endemic areas. The number of schools is not defined at the beginning as it is dependent on the number of high worm burden families that can be recruited in the school. We will continue to sample schools until we have recruited our target number of children, which is at least 100 families per country.

After screening children for intensity of infection, parents or guardians of children who meet inclusion criteria will be visited and invited to provide informed consent for themselves and their participating children. Children older than 10 years will be invited to provide assent. Each participant will be free to choose whether to join the study after the objectives, procedures, and potential risks and benefits of the study have been clearly explained to them using lay terms.

***Screening procedures.*** Schistosomiasis screening will be performed at schools by a team from TrypanoGEN+ together with the National Control Programs team. Children who agree to participate in the study will be tested for *S. mansoni* infection by detecting circulating cathodic antigen in their urine and *Schistosoma mansoni* eggs in their stools, using the Point Of Care Circulating Cathodic Antigen (POC-CCA) test ( van Dam *et al*., 2004) and the Kato Katz (KK) technique respectively ( World Health Organisation, 1994). Urine and stool will be collected in plastic collection tubes and bags. In each country, some samples may be kept in the institution which will conduct the research, i.e. University of Dschang (Cameroon samples), INRB (DRC) and University Jean Lorougnon Guédé University (Ivory Coast). All children will be treated with Praziquantel by the national Ministry of Health Mass Drug Administration teams according to the recommendation of the World Health Organization ( World Health Organisation, 2006). 

***Inclusion criteria***

1. Age between 5–15 years
2. Intensity of infection in the top 20% of positive children in their community
3. Have at least one parent available for inclusion in the study
4. Born in the community or spent at least a year in the community

***Exclusion criteria***

1. No parent available
2. Children positive for *S. haematobium* by urine filtration test or PCR
3. Treatment with praziquantel for schistosomiasis in the last six months

**Figure 1.**
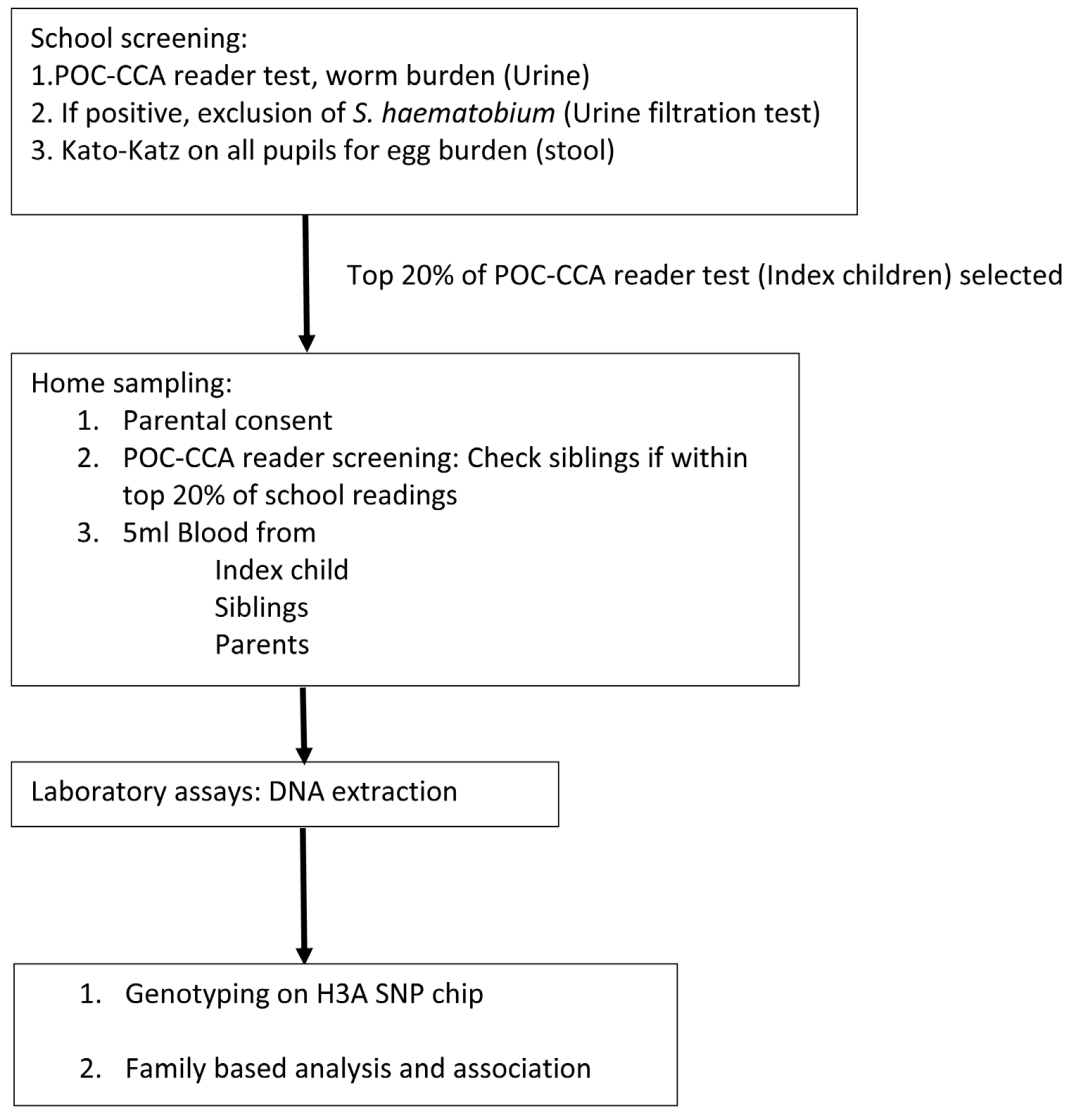
Study flow chart for family-based association study (FBAT) studies. POC-CCA=point of care-circulating cathodic antigen; PCR=polymerase chain reaction; DNA=deoxyribonucleic acid; SNP=single nucleotide polymorphisms.

***Procedures for the top 20% index children in families.*** The families of the children bearing high worm burden infections will be visited in their homes to meet their parent or guardian and inform them about the objectives and goals of the study. The head of the families that meet the inclusion and not the exclusion criteria will be invited to give their consent for their family to participate to the study. Adult participants will also be invited to give their consent and the index child and siblings will give their assent if over 10 years. Children, who are encountered in the home and have not been screened at school and meet the enrolment criteria, will be tested for inclusion in the study. Each person who gives consent or assent and meets the enrolment criteria will be asked to provide about 5ml of blood. This will be done in the home and samples will be kept at cold storage for transportation to the relevant laboratory; University of Dschang (Cameroon samples), INRB (DRC) and University Jean Lorougnon Guédé University (Ivory Coast). Figure 1 shows a flow-chart of the procedures for this study.

***Timelines.*** Timelines for the study are shown in Table 2. Initially, all work was projected to take 3 years. However, collections started in 2019 and may continue until 2023 due to restrictions imposed by the coronavirus disease 2019 (COVID-19) pandemic.

**Table 2.** Timeline for the study.

Event	Year1	Year 2	Year 3
Ethical clearance			
Administrative approval			
Sample collection			
Family/case-control inclusion			
DNA extraction			
Genotyping			
Data analysis			
Publication of manuscripts			

***Diagnosis***

Kato Katz test

Each participant will provide a stool sample for the Kato-Katz test (KK). The WHO approved standard KK test will be conducted ( Katz *et al*., 1972). To assure of the quality of the test, we will homogenize stool by mixing in a polythene bag to make the distribution more uniform. After this we will pick the stool using spatula from different areas of the stool sample and make the Kato Katz stool slide. We will take duplicate slides and have two separate readings of egg counts by technicians. The average of these readings is then taken as the eggs per gram.

This technique can be used to distinguish the different helminth species eggs ( *S. mansoni Ascaris lumbricoides*, *Trichuris trichiura*, hookworm and *Taenia* spp), which will be recorded separately.

Circulating cathodic antigen (CCA) test

The KK thick stool smear is the most common field technique for *S. mansoni* identification and quantification. This method is 100% specific, however it has poor sensitivity for detecting low intensity infections ( Colley *et al*., 2017). More recently the CCA that is produced by adult worms ( Nash & Deelder, 1985) and is detectable in blood and urine, has been used as the basis for a lateral flow test that can be used at the point of care ( Kittur *et al*., 2016; WHO, 2012; van Dam *et al*., 2004). The advantages of the POC-CCA test include its rapid procedure, easy handling under field conditions and minimal practical training requirements for its application ( Glinz *et al*., 2010). Several studies have shown that this test appears to be more sensitive than the KK technique ( Deelder *et al*., 1976; Kittur *et al*., 2016; Mnkugwe *et al*., 2020). Nevertheless, the specificity of POC-CCA is influenced by cross-reactivity with other helminth infections and, more importantly, may produce false positives with weak bands especially in areas of moderate and low prevalence and this can lead to overestimation of prevalence ( Colley *et al*., 2017). However in areas of high transmission, such as those where studies 1 and 2 are being undertaken, all trace positives should be treated as positives ( Colley *et al*., 2020). Although the abundance of CCA antigen is believed to be proportional to the number of worms present ( van Dam *et al*., 2004), the POC-CCA is marketed as a qualitative test for *S. mansoni* diagnosis and not for worm quantification. However, three studies have demonstrated that there is a semi-quantitative relationship between band intensity estimated visually and egg count on the Kato-Katz test ( Coulibaly *et al*., 2013; Dawson *et al*., 2013; Kittur *et al*., 2016). This has recently been improved with a set of 10 standards of reference cassettes (G-scores) with ink jet printed bands of increasing intensity ( Casacuberta-Partal *et al*., 2019). We have recently extended this method by using the ESEQuant LR3reader to read the intensity of the positive band. In field trials ESEQuant readings correlated well with estimates of band intensity using the reference cassettes developed by Casacuberta-Partal *et al.* (2019) with a Spearman rank correlation coefficient = 0.89 ( Mewamba *et al.*, 2021). We will therefore use the ESEQuant LR3 reader to objectively quantify band intensity on POC-CCA cassettes in a standardized way at all sites in Study 1 except the samples from Lake Victoria. The samples from the Uganda Lake Victoria site are already archived and worm burdens will be quantified using the more sensitive CAA assay.

Using the RDT-Reader for quantitative POC-CCA

The ESEQuant LR3 Gold reader (part number ESLR11-MB-6401 with a drawer inlay for the POC-CCA cassette part number ESLR05-MA-5167 DIALUNOX GmbH, Germany) will be used to measure reflective signals on POC-CCA cassettes. This gives an objective measure different from the subjective visual results. First, a method is developed on the reader to capture valid signals for the control line and test lines. This method will be deployed on subsequent RDT tests on this reader. A quantitative test will be done on the POC-CCA strip by spotting a drop of urine on the POC-CCA. The sample number will be pre-entered on the reader, and once 20 minutes elapse the cassette will be put into the reader and the method will be initiated to read the cassette. Cassettes that are not read at 20 minutes will be discarded and the test will be repeated. An OD signal indicating the intensity of signal will be shown. The intensity per sample is stored on the reader and can be downloaded to compare signal intensities across various samples.

Circulating anodic antigen (UCP-LF test)

The upconverted phosphor lateral flow (UCP-LF) is a highly sensitive assay that is used to detect the CAA antigen regurgitated by adult *Schistosoma* worms and is present in urine, plasma and serum ( van Dam *et al*., 2013). The CAA is much more abundant in plasma than CCA which in combination with its high specificity and fluorescence reading technology leads to a test with much higher sensitivity.

We will use the schistosome CAA assay that requires 20µl of plasma (SCAA20). Since the POC-CCA assay has only been used as a semi-quantitative test ( Coulibaly *et al*., 2013; Dawson *et al*., 2013; Kittur *et al*., 2016) we will validate the CCA test against the CAA test in 100 samples. A previous comparison of the two methods in a low endemicity community in Brazil found only modest correlation (Spearman’s rho = 0.24) between CAA and CCA readings which were classified as negative, trace or positive ( Sousa *et al*., 2019). In that study 36% of samples were positive by CAA, 12% by CCA and 1.6% by Kato-Katz demonstrating the relative sensitivity of the methods. CAA results will be compared against 100 CCA results for study 1 to validate CCA reader signals ability to pick out the top 20% of worm burden. We calculated the power to detect a correlation between CAA and CCA signal intensities using the pwr.r.test function in the R pwr library. 100 samples will have 80% power to detect a correlation (r> 0.28). We will also use the CAA test as the primary measure of worm burden for the Lake Victoria samples.

Human negative plasma will be spiked with a known concentration of CAA and dilutions made. A negative plasma sample that has not been spiked with CAA will be included as a negative control. Plasma samples and standards will be extracted with an equal volume of 4% w/v trichloroacetic acid, vortexed and incubated for 5 minutes. Of the resulting TCA soluble fraction, 20μL will be added to wells coated with UCP particles and anti-mouse monoclonal anti-CAA antibodies hydrated with 100ul of high salt lateral flow buffer. The wells will then be incubated for one hour at 37°C while shaking at 900rpm in a shaking incubator. After one hour, the lateral flow strips will be placed in UCP bound wells and the samples will be allowed to flow. The strips will be dried overnight and analysed using the Labrox Upcon scanner (serial number 2180023). The test line (T) signals will be normalized to the flow control (FC) signals of the individual strips and the results expressed as ratio values i.e. T/FC.

Urine filtration and PCR to exclude *S. haematobium*

Since *S. mansoni* CCA can also cross react with *S. haematobium* CCA, all CCA positive samples should be processed further to exclude *S. haematobium* co-infected samples. This will leave only the *S. mansoni* mono-infected samples for analysis. Urine filtration followed by microscopy will be used in the field as a screening test to exclude *S. haematobium* co-infected samples. All *S. haematobium* negative samples in the urine filtration will be further tested with *S. haematobium* specific PCR, a more sensitive test to exclude *S. haematobium* co-infection. Where urine filtration test is not available, all POC-CCA positive urine samples will directly undergo the PCR test without prior screening with the urine filtration test. The *S. haematobium* PCR works best on urine concentrates. *S. haematobium* positive samples will be identified by amplifying the repetitive *Dra1* sequence which is specific to S. *haematobium* using primers identified by Hamburger *et al*., 2001 ( Hamburger *et al*., 2001).

***Genotyping and association testing***

Candidate genes

Whilst many genes are known to participate in the response to schistosome infection ( Kamdem *et al*., 2018) there is a more limited set of genes that may have variants that contribute to the outcome of infection ( Mewamba *et al*., 2021a). A list of genes that will be tested is shown in Table 1. Candidate genes have been selected on three principles: 1) genes that have previously been shown to be associated with schistosomiasis in other studies; 2) genes in the QTL regions that have been reported to be involved in the response to schistosome infection; 3) genes that are involved in the Th _17_ pathway. We have previously shown that Th _17_ pathway genes are present in multiple quantitative trait loci and may contribute to the effect associated with these loci ( Mewamba *et al*., 2021a). Further justification and functional effects of selected SNPs for the genes selected is in the extended data ( Noyes, 2021b) and in ( Mewamba *et al*., 2021a).

Genotyping

DNA will be extracted from whole blood from index cases and their family members using the Qiagen mini kit (QIAamp DNA minikit catalogue number 51306, Qiagen, Germany) or the Quick-DNATM Miniprep Plus Kit (Catalogue Number: D4069, Zymo research, USA) according to manufacturer’s instructions. Genotyping will be done by Illumina (San Diego) on the Illumina H3Africa Pan African V2 chip ( The H3Africa Consortium *et al*., 2014) which contains approximately 2.5 million SNPs enriched for SNP that are informative in African populations.

Family based analyses

We will use family based analysis methods as reviewed by Ott *et al.* ( Ott *et al*., 2011). The method applied will depend on the structure of families collected. Here we describe one such method, the transmission disequilibrium test (TDT). The TDT assesses evidence of association between markers and schistosomiasis in the presence of linkage. The test statistic (also known as McNemar’s Test) is chi squared distributed $\chi^{2}=\frac{{\left(T-NT\right)}^{2}}{\left(T+NT\right)}$ where T is the number of times the allele of interest is transmitted from heterozygous parents to affected offspring and NT is the number of times that allele is not transmitted ( Spielman *et al*., 1993). We will use the family based association test (FBAT V1.4) implementation of the TDT ( Laird *et al*., 2000) to test each haplotype allele in each haplotype block for association with schistosomiasis. If there are sufficient families with more than one affected sib, then we will also use the affected sib-pair test in the same package. We will use the multi-locus transmission disequilibrium test (M-TDT) method ( Loucoubar *et al*., 2017) to search for evidence of additive and epistatic effects of multiple loci.

Meta-analysis

Initially data sets from each country with at least 100 families will be analysed independently to identify loci that are associated with populations in each country. Subsequently the data from all four participating countries will be combined in order to undertake a meta-analysis and identify loci that are of smaller effect than are detectable in local studies and that are also consistently associated with disease in multiple African countries. We will use R version 4 for plotting graphics and supplementary data, FBAT V1.4 will be used for fbat analysis and Plink V1.90 for transmission disequilibrium tests (TDT).

Association analysis with haplotypes

Family based methods for the discovery of linkage and association depend on being able to calculate the probability of the child genotypes given the genotypes of the parents. These calculations are only meaningful if there is more than one allele that the children might inherit from each parent, ie the parents need to be heterozygous in order for them to be informative at a particular locus.

Since SNP usually only have two alleles the frequencies of heterozygote parents will generally be low. For example, with the highest possible minor allele frequency (MAF) of 50%, 50% of parents will be heterozygous and only 25% of families will have two heterozygous parents. The frequency of heterozygotes can be increased by using SNP haplotypes that also have more alleles. In order to test the viability of using haplotypes, rather than SNP, for the association analysis we ran a simulation using 1,000 Genomes Project human sequence data ( Abecasis *et al*., 2012) to discover the empirical distribution of haplotype frequencies. Having identified 378 haplotype blocks within the 24 candidate genes we ran a power analysis to discover the proportion of loci where there was adequate power to discover different values of haplotype relative risk (supplementary text in extended data ( Noyes, 2021b). 

The power analysis found the number of families that would be required to detect haplotype relative risks (HRR) of 1.5, 2 and 3 with haplotype allele frequencies from 0.05 to 0.95 after correction for the 378 haplotype blocks being tested (extended data Figure 2A ( Noyes, 2021b). The analysis showed that 250 families would provide at least 80% power to detect a HRR of 2 for haplotypes with allele frequencies between 16% and 72%. At 80% of haplotype blocks over 50% of chromosomes were on a haplotype with a frequency in the informative range (extended data Figure 2B ( Noyes, 2021b). However, at 46 out of the 378 loci no haplotypes had allele frequencies in this range (extended data Figure 2C ( Noyes, 2021b) and therefore only large HRR (HRR > 2) are likely to be detectable at these loci. In summary 250 families will provide at least 80% power to detect HRR > 2 at the 60% of blocks that have haplotypes with allele frequencies between 16 and 72%. The number of alleles and allele frequencies in a haplotype block can be controlled by changing the block length. We used a standard block length of four SNP loci for our simulation but the optimum number for maximum potential power will vary between loci depending on allele frequencies and local linkage disequilibrium. Prior to running the association tests, we will use the genotype data to optimise the sets of haplotype blocks within each gene to maximise theoretical power to detect associations.

***Consent to publish.*** All authors will approve the final manuscript before publication. No institutional consent is required for publication.

***Data management.*** Study documents will be kept securely in the institution which will conduct the research, i.e. University of Dschang (Cameroon samples), INRB (DRC), Makerere University(Uganda samples) and University Jean Lorougnon Guédé University (Ivory Coast). These will be in physical lockable cabinets for forms and password protected databases with only study staff and investigators having access. All data will be anonymized before publication in appropriate scientific journals. Accompanying genotype data will also be anonymized and will be deposited in an open access depository.

Data will be collected in standardised forms as shown in supplementary data. It will be entered in Excel sheets and SQL database with range checks. The variables in the case report forms will be precoded for categorical variables to allow proper analysis. Physical data forms will be stored securely. Only the study investigators and project staff involved in generating the data will have access to the data.

***Dissemination of study outcomes.*** No actionable information is expected from the association analysis and individual results will not be returned to participants. General feedback will be shared with study participants and community leaders at public meetings and results shared with policy stakeholders including officials of the National Schistosomiasis Control Programs and Ministries of Health. Sponsors of the research will receive periodic reports on study progress. Analyses will be performed separately for each study population and published independently so that national authorities can have a clear understanding of the genetic risk factors for schistosomiasis in their countries. A meta-analysis of the combined data will also be undertaken and published separately. 

### Study 2: Case-control association study

***Study site: Lake Albert region, Uganda***. The major difference between Study 1 and Study 2 is the study design. They therefore share the schistosomiasis specific methods described above for study 1; the CCA test, Kato Katz, DNA extraction and genotyping and the panel of genes to be tested. Study 2 will differ from study 1 in recruitment procedures including inclusion criteria, definition of cases and controls and data analysis methods. Below we describe what will be the different in study 2.

***Recruitment and case definition.*** In the study site, the population will be screened using the CCA rapid test with results scored visually as trace, +1, +2, +3 (see section on CCA above) ( Casacuberta-Partal *et al*., 2019). All individuals will be invited to give a blood sample for DNA extraction and plasma for subsequent worm burden quantification by CAA.

Extreme phenotype sampling will be done, where only the genotypes of the top 25% and bottom 25% of worm burden will be analyzed as described by Li *et al.* ( Li *et al*., 2019). Genotyping only the participants with extreme phenotypes reduces the genotyping cost by 50% but has very little effect on power ( Li *et al*., 2019). To allow determination of these extreme phenotypes, worm burden will be determined on plasma using the CAA test in the laboratory at UVRI. The CAA test has been described above. Since the case-control design requires controls to be unrelated to the cases, the 25% of participants in the top and bottom tails of the CAA distributions will be included for genotyping. The samples will be genotyped using the H3A chip defined above. The genotype data will be used to filter out individuals who are first cousins or closer and share a phenotype to minimize bias.

After filtering, the target proportions of the total sample for cases and controls is expected to be at least 20% of the original population sampled. In a multiplicative model; a 20% fraction from each extreme phenotype would give 80% power to detect 35% increase in risk; for MAF 0.3 with sample size 300 (60 cases and 60 controls genotyped) ( Li *et al*., 2019). Most effects in the classical SM1 locus QTL have been shown to be co-dominant ( Abel *et al*., 1991; Marquet *et al*., 1996) and therefore a multiplicative model is appropriate.

**Figure 2.**
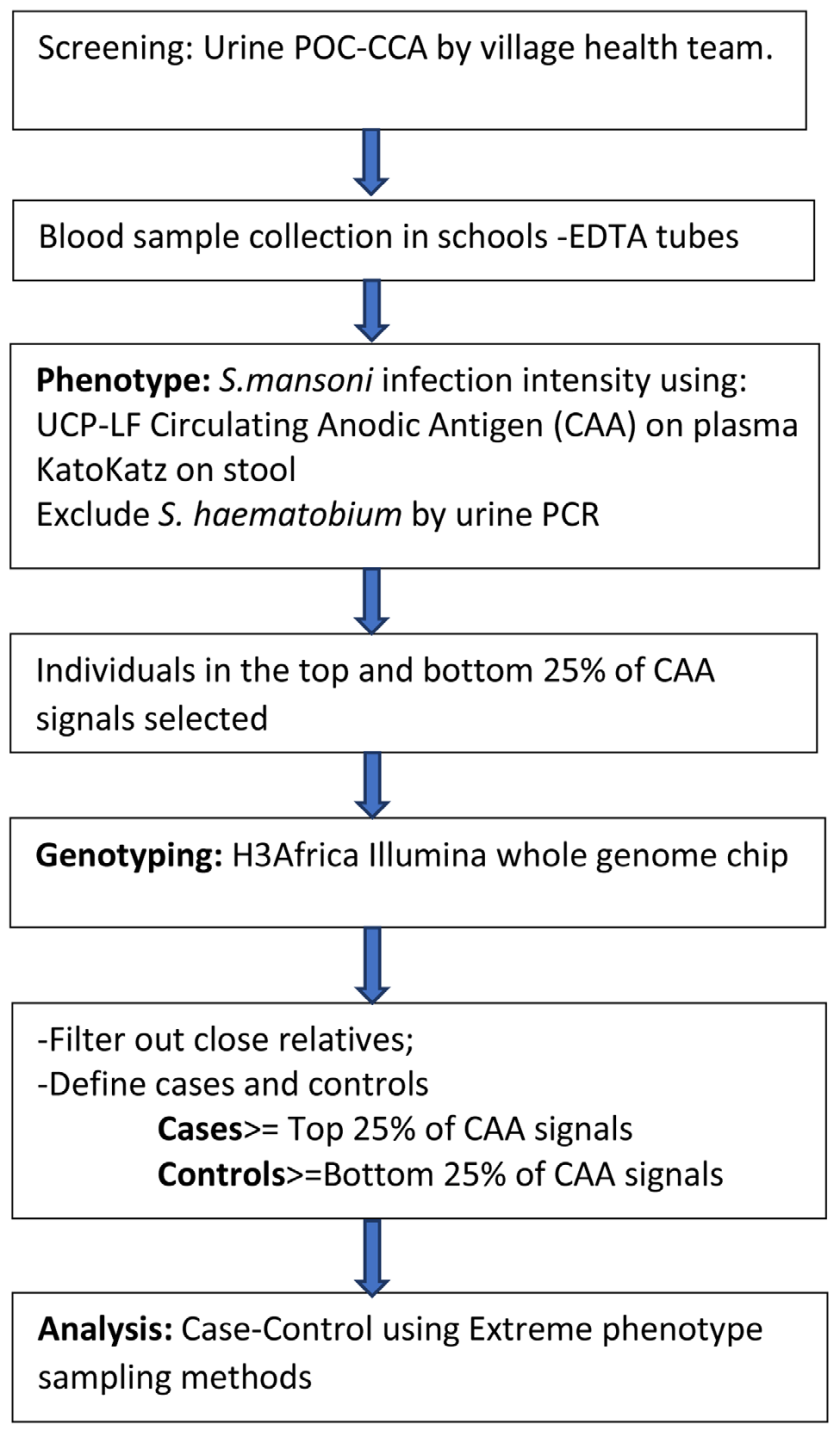
Study profile for the case-control study. POC-CCA=point of care-circulating cathodic antigen.

We will therefore aim to recruit at least 500 CAA positive children in Uganda. The case-control recruitment profile is shown in Figure 2

***Additional data recorded per participant.*** The following features will be recorded at the time of sample collection as potential covariates during analysis: age, sex, fever, diarrhea, infections with other parasitic worms will be detected by microscopy as part of the KK procedure, clinical observation for distended abdomen, Mid Upper Arm Circumference (MUAC) and body mass index,BMI (weight divided by the square of height in metres). The case report is provided as part of the extended data ( Noyes, 2021a).

***Data analysis.*** A candidate gene case control linear regression analysis will be undertaken in Plink v1.9 and R 4.0, with haplotypes as the independent variable and CAA as the dependent variable. Results will be controlled for population structure using principal components also calculated in Plink v1.9 as covariates. In this sampling design, it is critical to control for population structure ( Panarella & Burkett, 2019), and genotyping on the whole genome H3A chip will provide sufficient data for a calculation of principal components for this purpose. Age, sex, MUAC and BMI will also be used as additional covariates. Procedures for the KK test will be the same as those of study 1.

***Dissemination of study outcomes.*** Study participants will immediately receive their results for testing of schistosomiasis and will be advised on control measures by the health team. Since the national control programs are also involved in the study, they will have access to the results of the study for use in programmatic operations and policy decisions.

### Selection of candidate genes to be tested

The set of candidate genes that we will test ( Table 1) is based on our recent review of all previous studies of genetic variants associated with schistosomiasis infection intensity ( Mewamba *et al*., 2021a). We included candidate genes based on three sets of overlapping criteria:

1) Eleven genes that have been found associated with schistosomiasis in other populations ( *IFNG, IL10, IL13, IL4, IL5, STAT6, CTLA4, FCN2, COLEC11, ABO, RNASE3*)
2) Five genes in schistosomiasis quantitative trait loci (QTL) that are known to participate in the response to schistosomiasis infection but have not previously been tested in candidate gene studies ( *IL17A, IL17B, IL17F, IL6R, IL12B*)
3) Eight genes in the Th _17_ pathway ( *IL1A, IL1B, TGFB1, IL6, IL21, IL23A, IL25, IL17RA*)

SNP in only four of the eleven previously tested genes in Table 1 have been found associated with infection with schistosomes in more than one population, these genes are indicated by multiple citations in Table 1. It is important to replicate the findings for the other genes since the earlier studies were generally small and candidate gene studies are known to be poorly reproducible ( Hirschhorn *et al*., 2002). Amino acid changes and functional effects of selected SNP in these genes are in Supplementary_tableVEP1.tab ( Noyes, 2021b).

Four quantitative trait loci (QTL) associated with *S. mansoni* egg count have been discovered ( Marquet *et al*., 1996; Marquet *et al*., 1999; Müller-Myhsok *et al*., 1997; Zinn-Justin *et al*., 2001). These QTL were discovered before the human genome provided lists of genes in these regions. We systematically scanned these regions for genes that have been studied in the context of schistosomiasis ( Mewamba *et al*., 2021a). Genes in QTL with strong evidence for involvement in the response to infection have been included in this study. These genes are annotated with the QTL location in Table 1.

Three of the four QTL regions contained genes in the Th _17_ pathway. The Th _17_ system has two main functions: it regulates the clearance of extracellular pathogens, it also helps the B cells to induce tissue inflammation ( Patel & Kuchroo, 2015). Although the Th _17_ system is known to be involved in the response to schistosomiasis ( Larkin *et al*., 2012; Mbow *et al*., 2013), no studies of the genetics of Th _17_ pathway genes in human schistosomiasis have been conducted so far. We therefore included Th _17_ genes in the QTL regions in our study ( Table 1) as well as important Th _17_ genes that were outside the QTL ( *IL1A, IL1B*, *IL17RA, IL21, IL23, IL25, IL6, TGFB1*).

By testing for association with schistosomiasis in four different populations we will be able to identify genes which have variants which contribute to the heaviest worm burdens across Africa. Other genes may be of local importance or previously observed associations might be false positives as a consequence of sampling from stratified populations. Genetic data has recently been used to compile risk scores for developing schistosomiasis related sever liver disease ( Dessein *et al*., 2020). With the data from this study, it may be possible to develop a similar risk score for acquiring high worm burdens. 

## Discussion

There is abundant evidence for genetic contributions to the risk of acquiring schistosomiasis and to having particularly heavy worm burdens ( Dessein *et al*., 2020; Mewamba *et al*., 2021a). Some of this evidence has come from candidate gene studies that tested for association with genes that were known to be involved in the response to infection, but these studies have rarely been replicated and, where they have, the results have often been inconsistent. Other evidence has come from mapping studies that have identified QTL, regions of the genome that contain one or more genes with variants that change the risk of schistosomiasis. However, in most cases, no genes within these regions have been tested for association with disease. We have systematically surveyed these genes for plausible candidate genes which are known to participate in the response to infection ( Mewamba *et al*., 2021a) and observed that Th17 pathway genes were found within the majority of these regions.

We will genotype the samples on the H3Africa Whole Genome SNP chip making it possible to scan for associations with any gene in the genome in a Genome Wide Association Study (GWAS). However large numbers of samples are required for a GWAS to compensate for the large number of tests being conducted and, since we only expect to collect 100 families per population for family-based analyses and 100 cases and 100 controls for the case control study, we would only have the power to detect exceptionally large relative risks in a GWAS. By declaring the list of candidate genes in advance of analyzing the data we can establish a much more limited set of hypotheses which we will have the power to test with the number of samples available.

## Study status

The study is still recruiting participants. The current progress is: Cameroon (100 families consisting of 411 individuals); Ivory Coast (10 families consisting of 39 individuals); Democratic Republic of Congo (14 families consisting of 40 individuals); Uganda (LaVIISWA; previous family based collection, 40 families; Albertine case control collection: 600 individuals).

## Data availability

### Underlying data

No data are associated with this article.

### Extended data

Harvard Dataverse: Consent forms and CRFs for family based or case control schisto studies. https://doi.org/10.7910/DVN/6OLLGW ( Noyes, 2021a).

This project contains the following extended data:

- CAM_autorisation recherche schisto.pdf (Cameroon health ministry authorisation for research)
- CAM_Clairence éthique schistosomiase.pdf (Cameroon ethics review clearance)
- CAM_Consent form_Anglais.pdf (Cameroon consent form in English)
- CAM_Consent form_french.pdf (Cameroon Consent form in French)
- CAM_Information sheet.pdf (Cameroon information sheet)
- CIV_Assent form.docx (Ivory Coast Assent form English)
- CIV_Consentements_Projet TrypanoGen+.docx (Ivory Coast information sheet, consent forms in French)
- CIV_Informed Consent Form.docx (Ivory Coast Consent form in English)
- DRC_collective_consentform_and_CRFs.docx (Democratic Republic of Congo Consent form and case report form)
- DRC_Fiche de Consentement et Information.docx (DRC information sheet)
- DRC_Fiche de Consentement Personnel.docx (DRC personal consent form)
- UGA_LaVIISWA_consent_form.pdf (Uganda LaVIISWA study consent form: Refer for future use allowing genetic studies)
- UGA_SCHISTO Assent.pdf (Uganda assent form for the case-control study)
- UGA_Schisto Consent.pdf (Uganda case control study consent form)
- UGA_SCHISTO CRF.pdf (Uganda case control study case report form)
- UGA_Schisto Sample Storage.pdf (Uganda case control study sample storage consent)

Harvard Dataverse: Haplotype Genotyping. https://doi.org/10.7910/DVN/OVQPCO ( Noyes, 2021b). 

This project contains the following extended data:

- Amino acid changes and biological consequences associated with SNP selected from genes in Table 1
  ○ Supplementary_tableVEP1.xlsx Selected SNP functional effects from Variant effect predictor tool http://grch37.ensembl.org/info/docs/tools/vep/
- Files for modelling the effect of using haplotypes for transmission disequilibrium test
  ○ SupplementaryMaterial_Association_analysis_with_haplotypes.pdf (Description of association analysis with haplotypes and the power to detect different haplotype relative risks given different haplotype allele frequencies)
  ○ SupplementaryTables_1KG_African_haplotype_blocks.xlsx (Excel file containing lists of haplotype blocks identified in candidate genes in One Thousand genomes African samples, together with the number of samples required to have 80% power to detect Haplotype relative Risk of 1.5 or 2.0 or 3.0. Also, a worksheet with details of each haplotype in each block)
- Software
  ○ ReadMe.txt (Description of how to run the pipeline)
  ○ makeHaplotypes.pl (perl script called by runBigLD.sh to obtain the SNP in each haplotype block, identify all the haplotypes in a block and count their frequencies. It outputs Summary.HapStats.1kg.h3a.POP.txt containing summary statistics on each haplotype block in each gene in a population and All.HapsStats.1kg.h3a.POP.txt containing the sequence of each haplotype in each haplotype block in each candidate gene and counts of observations of each haplotype. POP is a population name)
  ○ plotBlocks.R (Rscript to make plots of haplotype blocks for each 1000 genomes African population)
  ○ replaceFamIds.pl (Perl script to modify 1000 genome sample ids so that they ids include a three-letter population identifier. Uses ids and population names in OneKg.pops. Takes a fam file with 1000 genome ids as input parameter)
  ○ runBigLD.R (Rscript that uses the BigLD R package to identify haplotype blocks and output a linkage disequilibrium heatmap and a text file of haplotype block co-ordinates. The text file is subsequently deleted)
  ○ runBigLD.sh (Wrapper shell script that prepares input files for runBigLD.R, calls runBigLD.R and also calls makeHaplotypes.pl to process runBigLD.R output)
- Data files used in input
  ○ OneKg.pops (Text file with all 1000 genome ids and the populations to which they belong. Used by replaceFamids.pl)
  ○ exons.ensGRCh37.txt (Co-ordinates of exons for use by plotBlocks.R)
  ○ getExons.sh (shell script to extract exons for just genes of interest from a larger mart_export.txt (not provided) file which should be downloaded from Biomart. Note the script makes assumptions about the columns in which data will be found and these should be adjusted according to the mart_export.txt file used)
  ○ SchistoCandGenes.1kg.h3a.bed (Plink bed file with genotypes of SNP that are present in both the 1,000 genomes dataset and the Illumina H3Africa genotyping chip and that are in the candidate genes analysed)
  ○ SchistoCandGenes.1kg.h3a.bim (Plink bim file with identifiers of SNP that are present in both the 1,000 genomes dataset and the Illumina H3Africa genotyping chip and that are in the candidate genes analysed)
  ○ SchistoCandGenes.1kg.h3a.fam (Plink fam file with identifiers for 1000 genomes African samples)
- Output from simulations for each country and combined
  ○ All.HapsStats.1kg.h3a.ALL.txt (Sequence of each haplotype in each haplotype block in each candidate gene and counts of observations of each haplotype for all populations (GWD, MSL, YRI, ESN, LWK))
  ○ All.HapsStats.1kg.h3a.ESN.txt (Sequence of each haplotype in each haplotype block in each candidate gene and counts of observations of each haplotype for ESN population)
  ○ All.HapsStats.1kg.h3a.GWD.txt (Sequence of each haplotype in each haplotype block in each candidate gene and counts of observations of each haplotype for GWD population)
  ○ All.HapsStats.1kg.h3a.LWK.txt (Sequence of each haplotype in each haplotype block in each candidate gene and counts of observations of each haplotype for LWK population)
  ○ All.HapsStats.1kg.h3a.MSL.txt (Sequence of each haplotype in each haplotype block in each candidate gene and counts of observations of each haplotype for MSL population)
  ○ All.HapsStats.1kg.h3a.YRI.txt (Sequence of each haplotype in each haplotype block in each candidate gene and counts of observations of each haplotype for YRI population)
  ○ GeneHapBlockPlots.pdf (Output from plotBlocks.R)
  ○ Heatmaps.tif.tar.gz (Heatmaps showing linkage disequilibrium and haplotype blocks within each gene. The heatmaps are output from the runBigLD.R Rscript)
  ○ Summary.HapStats.1kg.h3a.ALL.txt (Summary statistics on each haplotype block in all populations (GWD, MSL, YRI, ESN, LWK) for each gene for all populations)
  ○ Summary.HapStats.1kg.h3a.ESN.txt (Summary statistics on each haplotype block in each gene for ESN population)
  ○ Summary.HapStats.1kg.h3a.GWD.txt (Summary statistics on each haplotype block in each gene for GWD population)
  ○ Summary.HapStats.1kg.h3a.LWK.txt (Summary statistics on each haplotype block in each gene for LWK population)
  ○ Summary.HapStats.1kg.h3a.MSL.txt (Summary statistics on each haplotype block in each gene for MSL population)
  ○ Summary.HapStats.1kg.h3a.YRI.txt (Summary statistics on each haplotype block in each gene for YRI population)

Data are available under the terms of the Creative Commons Zero "No rights reserved" data waiver (CC0 1.0 Public domain dedication).
