# Supplementary material for: Candidate gene family-based and case-control studies of susceptibility to high Schistosoma mansoni worm burden in African children: a protocol
Source: AAS Open Res. 2021 Dec 15;4:36. Originally published 2021 Jun 29. [Version 2] doi: 10.12688/aasopenres.13203.2 (PMC8861467; doi:10.12688/aasopenres.13203.2)
Supplement: Peer review history

# Peer review: Reviewer response for version 2

**DOI:** 10.21956/aasopenres.14458.r29061

**Published:** 2022-02-21

**Authors:** Samuel K. Armoo

**Affiliations:** 1 Biomedical and Public Health Research Unit, Water Research Institute, Council for Scientific and Industrial Research, Accra, Ghana

Approved.

Is the study design appropriate for the research question?

Yes

Is the rationale for, and objectives of, the study clearly described?

Yes

Are sufficient details of the methods provided to allow replication by others?

No

Are the datasets clearly presented in a useable and accessible format?

Not applicable

Reviewer Expertise:

NA

I confirm that I have read this submission and believe that I have an appropriate level of expertise to confirm that it is of an acceptable scientific standard.

# Peer review: Reviewer response for version 1

**DOI:** 10.21956/aasopenres.14319.r28888

**Published:** 2021-10-20

**Authors:** Samuel K. Armoo

**Affiliations:** 1 Biomedical and Public Health Research Unit, Water Research Institute, Council for Scientific and Industrial Research, Accra, Ghana

**Summary:**

 This manuscript describes the relationship between underlying human host genotypes and the level of susceptibility or otherwise to high *Schistosoma mansoni *worm burdens. The authors tested 24 candidate genes in four endemic African countries and found an over-representation of some haplotypes in study participants with high worm burdens. This manuscript falls within the scope of this journal, but there should be some adjustments to make it suitable for indexing.

**Specific comments: **

- Table 1: Provide any amino acid changes from SNPs and their proposed functional effects.

**Sampling procedure:**

- **Inclusion Criteria**: Specify whether the duration of stay in the community is an inclusion criterion. If not, why? In addition, does the child need to be born in the community? If not, why? 

**Diagnosis:**

***Kato-Katz:***

1. Which quality control procedures were used for the Kato-Katz test?  
2. Why not use *S. mansoni *eggs per gram of stool for quantification of worm burdens?

***CCA:***

1. Armoo *et al.* (2020 ^1^) detected low specificity of the POC-CCA assay, when trace bands were included in high transmission areas in Ghana. Specificity increased significantly (from 12.9 to 76.5%) when trace bands were excluded. So kindly consider the role of trace bands in this study. 
2. CCA should be complemented by Kato-Katz or qPCR to determine worm burden phenotype. 
3. Which quality control procedures were used for the CCA and CAA assays? 

Is the study design appropriate for the research question?

Yes

Is the rationale for, and objectives of, the study clearly described?

Yes

Are sufficient details of the methods provided to allow replication by others?

No

Are the datasets clearly presented in a useable and accessible format?

Not applicable

Reviewer Expertise:

Medical parasitology and molecular genetics of neglected tropical diseases.

I confirm that I have read this submission and believe that I have an appropriate level of expertise to confirm that it is of an acceptable scientific standard, however I have significant reservations, as outlined above.

## References

[1] : Detecting Schistosoma mansoni infections among pre-school-aged children in southern Ghana: a diagnostic comparison of urine-CCA, real-time PCR and Kato-Katz assays. *BMC Infect Dis* .2020;20(1) : 10.1186/s12879-020-05034-2 301 10.1186/s12879-020-05034-2 32321418PMC7178570

# Response

**Published:** 2021-12-02

**Authors:** Oscar Nyangiri

**Affiliations:** Makerere University, Uganda

**Summary:**

**This manuscript describes the relationship between underlying human host genotypes and the level of susceptibility or otherwise to high *Schistosoma mansoni *worm burdens. The authors tested 24 candidate genes in four endemic African countries and found an over-representation of some haplotypes in study participants with high worm burdens. This manuscript falls within the scope of this journal, but there should be some adjustments to make it suitable for indexing.**

 We clarify that this is a protocol describing how we will test these 24 candidate genes and that we do not yet have results.

**Specific comments: **

- **Table 1: Provide any amino acid changes from SNPs and their proposed functional effects.**

We used the tool variant effect predictor here http://grch37.ensembl.org/info/docs/tools/vep/ to query the SNP IDs (rsIDs). In the legend of table 1 we add a reference to supplementary Supplementary_tableVEP1.xls ( Noyes, 2021b)

 Amino acid changes are under column header “Amino_acids” while functional effects are under column headers

  “Consequence”, which gives the position whether upstream, downstream of gene, regulatory, in gene whether missense or synonymous

 “BIOTYPE” which allows for a check whether the variant is in protein coding region or not

 “SIFT” and “PolyPhen” which gives a score of whether the variant is tolerated, benign or deleterious,

  “CLIN_SIG” which is clinical significance corresponding to whether the variant is a risk factor or protective

**Sampling procedure:**

- **Inclusion Criteria: Specify whether the duration of stay in the community is an inclusion criterion. If not, why? In addition, does the child need to be born in the community? If not, why? **

Thanks for pointing out this omission. We aim to recruit members resident in the communities and therefore duration of stay should be in the last 1 year. We have added this under the section “inclusion criteria”  

 Additionally, members born in the community are bona fide community members and should therefore be included.

 In summary, the rationale for adding these criteria: To avoid spurious results from imported infections or uninfected individuals, the members should have homogenous exposure to the risk in this community. Therefore, they should either be born in the community or if not; have spent a considerable amount of time in the community to be considered members with the same exposure as the rest of the community members.

**Diagnosis:**

***Kato-Katz:***

1. **Which quality control procedures were used for the Kato-Katz test?  **

We will homogenize stool by mixing in a polythene bag to make the distribution more uniform.  After this we will pick the stool using spatula from different areas of the stool sample and make the Kato Katz stool slide. We will take duplicate slides and have two separate readings of egg counts by two technicians.  The average of these readings is then taken as the eggs per gram.

**2. Why not use *S. mansoni *eggs per gram of stool for quantification of worm burdens?**

 We will indeed use two parallel tests: KK eggs per gram and POC CCA. CAA will also be used for all Uganda samples. Although they do not measure the exact worm/egg antigens or actual eggs observed, there is some concordance between these tests as published for CCA and KK (Coulibaly et al., 2013; Dawson et al., 2013; Kittur et al., 2016) and for KK and CAA (van Dam et al., 2013). The order of sensitivity is CAA>CCA>KK. Since our study is primarily interested in the top 20% of worm burden, the most sensitive tests, reader based POC-CCA and CAA are chosen to maximize the ability to discriminate between samples and allow a better estimation of genotypes over-represented in extreme phenotypes of the top 20% worm burden

 In the introduction under the section of how the proposed study aims to improve on previous studies, we state that CAA/CCA are more reproducible than the Kato-Katz method. “The Kato-Katz method of counting eggs in stool samples is known to lack sensitivity and this is compounded by day to day variation in egg excretions and uneven distribution of eggs across an individual stool (Kongs et al., 2001).”

***CCA:***

1. **Armoo *et al.* (2020 ^1^ ) detected low specificity of the POC-CCA assay, when trace bands were included in high transmission areas in Ghana. Specificity increased significantly (from 12.9 to 76.5%) when trace bands were excluded. So kindly consider the role of trace bands in this study. **

Thanks for this observation. We note from Armoo et al. (2020 ^1^) that inclusion of trace bands reduced specificity. The gold standard to assess specificity in Armoo et al, 2020 was the combination of POC-CCA and real time PCR Taqman.

 To account for this apparent lack of specificity from POC-CCA trace bands; our study uses a POC-CCA reader to convert the signal from the all bands in the POC-CCA test to optical intensity (see section “Using the RDT-Reader for quantitative POC-CCA”). In this way, we have a continuity from barely positive to highly positive.  The lower limit of detection on the POC-CCA reader is 40mV of reflected optical density, which detects very faint bands. We consider these trace bands in the bottom 20% of worm burden.

 We also use CAA the most sensitive method as a gold standard to establish whether CCA trace was actually positive. Our CAA assay has a sensitivity of up to 30 pg/mL of the circulating anodic antigen.

 We are also sampling in high transmission areas. According to Colley et al. 2020 trace bands are generally true positives in high transmission areas but are more likely to be false positives in low transmission areas

 It is important to note in the family-based studies that we are only genotyping the individuals who have the heaviest 20% of worm burdens and none of these will have trace CCA readings.

**2. CCA should be complemented by Kato-Katz or qPCR to determine worm burden phenotype.**

 In the section “Diagnosis” we describe all the methods we will use for diagnosis of schistosomiasis. Here, we describe Kato-Katz, CCA, a reader based CCA assay and CAA. We have therefore complemented CCA with KK.

 The CAA test will be done for a subset of those tested by both KK and reader based CCA assay to check for concordance between these tests.

 At least one of either KK or CAA have been used previously for similar analyses of genetic conditioning of egg or worm burden phenotype (Marquet et al., 1996; Marquet et al., 1999; Müller-Myhsok et al., 1997; Zinn-Justin et al., 2001). Mewamba et al 2021 has also shown how the reader-based method correlates with KK and can be used quantitatively.

 Regarding qPCR, it is a helpful additional method but at this time we are not able to do qPCR because of resource constraints.

**3. Which quality control procedures were used for the CCA and CAA assays?**

 The CCA was done in two ways

1.
  1. scoring the reading by eye from negative (0), trace (1), intermediate (2) and strong (3) as described in “Circulating Cathodic antigen (CCA) test” and using the G-scores as a standardization scoring system (as explained in Mewamba et al 2021)
  2. Using the reader to pick the optical intensity of the bands as described in section “Using the RDT-Reader for quantitative POC-CCA”

In our analysis we will compare the two readings, where we expect to see a concordance between the qualitative readings by eye with those from the reader. We have observed this already in one site, Cameroon (Mewamba et al 2021).

 We will use CAA on a subset of samples to validate our CCA results. CAA comes with standards from the manufacturer which we use to calibrate our readings.

 In addition to these, we will compare our readings with KK to check for trends. Particularly we will be interested to see if top/bottom samples in CAA are also matching respective top/bottom samples in CCA and KK.

**References**

1. Armoo S, Cunningham LJ, Campbell SJ, Aboagye FT, et al.: Detecting Schistosoma mansoni infections among pre-school-aged children in southern Ghana: a diagnostic comparison of urine-CCA, real-time PCR and Kato-Katz assays. *BMC Infect Dis*. 2020; **20** (1): 301 PubMed Abstract | Publisher Full Text
2. Colley DG, King CH, Kittur N, Ramzy RMR, Secor WE, Fredericks-James M, Ortu G, Clements MN, Ruberanziza E, Umulisa I, Wittmann U, Campbell CH. Evaluation, Validation, and Recognition of the Point-of-Care Circulating Cathodic Antigen, Urine-Based Assay for Mapping *Schistosoma mansoni* Infections. Am J Trop Med Hyg. 2020 Jul;103(1_Suppl):42-49. doi: 10.4269/ajtmh.19-0788. PMID: 32400347; PMCID: PMC7351311.
3. Coulibaly, Jean T., Yves K. N’Gbesso, Stefanie Knopp, Nicaise A. N’Guessan, Kigbafori D. Silué, Govert J. van Dam, Eliézer K. N’Goran, and Jürg Utzinger. ‘Accuracy of Urine Circulating Cathodic Antigen Test for the Diagnosis of Schistosoma Mansoni in Preschool-Aged Children before and after Treatment’. *PLoS Neglected Tropical Diseases* 7, no. 3 (2013): e2109. https://doi.org/10.1371/journal.pntd.0002109.
4. Dawson, Emily M., José C. Sousa-Figueiredo, Narcis B. Kabatereine, Michael J. Doenhoff, and J. Russell Stothard. ‘Intestinal Schistosomiasis in Pre School-Aged Children of Lake Albert, Uganda: Diagnostic Accuracy of a Rapid Test for Detection of Anti-Schistosome Antibodies’. *Transactions of the Royal Society of Tropical Medicine and Hygiene* 107, no. 10 (October 2013): 639–47. https://doi.org/10.1093/trstmh/trt077.
5. Kittur, Nupur, Jennifer D. Castleman, Carl H. Campbell, Charles H. King, and Daniel G. Colley. ‘Comparison of Schistosoma Mansoni Prevalence and Intensity of Infection, as Determined by the Circulating Cathodic Antigen Urine Assay or by the Kato-Katz Fecal Assay: A Systematic Review’. *The American Journal of Tropical Medicine and Hygiene* 94, no. 3 (March 2016): 605–10. https://doi.org/10.4269/ajtmh.15-0725.
6. Kongs, A., G. Marks, P. Verlé, and P. Van der Stuyft. ‘The Unreliability of the Kato-Katz Technique Limits Its Usefulness for Evaluating S. Mansoni Infections’. *Tropical Medicine & International Health* 6, no. 3 (2001): 163–69. https://doi.org/10.1046/j.1365-3156.2001.00687.x.
7. Marquet, S., L. Abel, D. Hillaire, H. Dessein, J. Kalil, J. Feingold, J. Weissenbach, and A. J. Dessein. ‘Genetic Localization of a Locus Controlling the Intensity of Infection by *Schistosoma Mansoni* on Chromosome 5q31-Q33’. *Nature Genetics* 14, no. 2 (October 1996): 181–84. https://doi.org/10.1038/ng1096-181.
8. Marquet, S., L. Abel, D. Hillaire, and A. Dessein. ‘Full Results of the Genome-Wide Scan Which Localises a Locus Controlling the Intensity of Infection by *Schistosoma Mansoni* on Chromosome 5q31-Q33’. *European Journal of Human Genetics: EJHG* 7, no. 1 (January 1999): 88–97. https://doi.org/10.1038/sj.ejhg.5200243.
9. Müller-Myhsok, B., F. F. Stelma, F. Guissé-Sow, B. Muntau, T. Thye, G. D. Burchard, B. Gryseels, and R. D. Horstmann. ‘Further Evidence Suggesting the Presence of a Locus, on Human Chromosome 5q31-Q33, Influencing the Intensity of Infection with Schistosoma Mansoni’. *American Journal of Human Genetics* 61, no. 2 (August 1997): 452–54. https://doi.org/10.1016/S0002-9297(07)64073-7.
10. Zinn-Justin, A., S. Marquet, D. Hillaire, A. Dessein, and L. Abel. ‘Genome Search for Additional Human Loci Controlling Infection Levels by *Schistosoma Mansoni*’. *The American Journal of Tropical Medicine and Hygiene* 65, no. 6 (December 2001): 754–58. https://doi.org/10.4269/ajtmh.2001.65.754.
11. Mewamba, Estelle Mezajou, Arnol Auvaker Zebaze Tiofack, Cyrille Nguemnang Kamdem, Romuald Isaka Kamwa Ngassam, Mureille Carole Tchami Mbagnia, Oscar Nyangiri, Harry Noyes, Hilaire Marcaire Womeni, Flobert Njiokou, and Gustave Simo. ‘Field Assessment in Cameroon of a Reader of POC-CCA Lateral Flow Strips for the Quantification of Schistosoma Mansoni Circulating Cathodic Antigen in Urine’. *PLoS Neglected Tropical Diseases* 15, no. 7 (July 2021): e0009569. https://doi.org/10.1371/journal.pntd.0009569.

# Peer review: Reviewer response for version 1

**DOI:** 10.21956/aasopenres.14319.r28821

**Published:** 2021-08-23

**Authors:** Severin Donald Kamdem

**Affiliations:** 1 Departement of pathology, School of Medicine, University of Utah, Salt Lake City, UT, USA

The manuscript *Candidate gene family-based and case-control studies of susceptibility to high Schistosoma mansoni worm burden in African children: a protocol [version 1; peer review: awaiting peer review]* by Nyangiri *et al. *aims to test 24 candidate genes, mainly in the Th2 and Th17 pathways, for association with *S.* *mansoni *infection intensity in four African countries (Ivory Coast, Cameroon, Uganda and Democratic Republic of Congo), using family based and case-control approaches. Regarding the dissimilarity observed in Schistosomiasis infection and related morbidity, this study is relevant for the field and could help for a better management of Schistosomiasis in endemic areas.

 The authors have done a preliminary work by reviewing all genetic factors that control Schistosomiasis infection (Mewamba *et al.* 2021, Frontiers in Immunology) and seem to have the baseline knowledge to conduct the proposed study. The objectives of the study are clearly established and the description of the Methodology appropriate. The respect of the ethic is well described and the authorization needed gathered. The proposed statistics and meta-analysis are comprehensive.

 However, the following suggestions will help improve the overall quality of the results.

 Since the authors intend to include school children, they will consider obtaining authorization from Ministries of Education.

 In addition to CCA and CAA, the authors should consider taking several stool samples (2 to 3) from consecutive days for KK analysis for an increased sensitivity using the resulting geometric mean of burden intensity. In fact, the CCA test could easily lead to false positive results in endemic areas, and the CAA is a good correlate for worm burden and not of eggs burden (as CAA levels indeed reflect dynamics of worm burdens and not of egg excretion or worm fecundity). KK remains the appropriate technique to accurately determine the egg burden. Of note, Schistosomiasis pathology is eggs (and not worms) driven. CCA and CAA can be useful in determining controls (true negative with all techniques) in the case-controls studies in Uganda.

 The authors propose to use the top 25% positive participants as participants with high burden. Considering the WHO classification for *S. mansoni* infection (light < 100; 100 < Moderate <400 or heavy > 400), what would be the contingency plan if participants in the top 25% have a burden lower than 400 EPG? Or 100EPG?

 The Figure 1 is quite confusing. The authors should consider revising it. Also, excluded samples should be mentioned. In addition, participants should be screened for clinical signs of anemia and excluded prior to blood collection.

 The authors should consider screening for other confections such as malaria etc. - generally co-endemic in these regions, as co-infections could affect the susceptibility of Schistosomiasis infection. Risk factors of susceptibility/Resistance to Schistosomiasis infection will be valuable information to have.

 It is not mentioned if the participants will be treated with Praziquantel at the end of the study.

 For the case-control study, the authors should correct for socio-demographic data to ensure minimal variation across the groups. For example, to fully attribute the difference in infection intensity to the genetics, the authors should make sure factors like age; gender; BMI etc. are not different between cases and controls. This will aid in minimizing confounding factors.

Is the study design appropriate for the research question?

Yes

Is the rationale for, and objectives of, the study clearly described?

Yes

Are sufficient details of the methods provided to allow replication by others?

Partly

Are the datasets clearly presented in a useable and accessible format?

Not applicable

Reviewer Expertise:

Infectious Diseases and Fibrosis

I confirm that I have read this submission and believe that I have an appropriate level of expertise to confirm that it is of an acceptable scientific standard.

# Response

**Published:** 2021-11-15

**Authors:** Oscar Nyangiri

**Affiliations:** Makerere University, Uganda

We appreciate this review and the useful comments received which will help to improve our manuscript. Below, we seek to address each of the concerns; with concerns and in bold and response in normal type font.

**Since the authors intend to include school children, they will consider obtaining authorization from Ministries of Education.**

 We considered the ethical procedures in each country, and in the section “Ethical approval and consent to participate” we describe what each country is doing which has been accepted by the ethical review committee. We already include district level school authorities in our authorization and community engagement work. We agree that the suggestion to involve the Ministry of Education is useful and we will suggest to our community engagement team to include the Ministry of Education where it is not currently included. Therefore in this section we add the statement “In all countries, since schools are involved, we will consult the ministry of education in conducting the study.”

**In addition to CCA and CAA, the authors should consider taking several stool samples (2 to 3) from consecutive days for KK analysis for an increased sensitivity using the resulting geometric mean of burden intensity. In fact, the CCA test could easily lead to false positive results in endemic areas, and the CAA is a good correlate for worm burden and not of eggs burden (as CAA levels indeed reflect dynamics of worm burdens and not of egg excretion or worm fecundity). KK remains the appropriate technique to accurately determine the egg burden. Of note, Schistosomiasis pathology is eggs (and not worms) driven. CCA and CAA can be useful in determining controls (true negative with all techniques) in the case-controls studies in Uganda.**

 Although we agree that eggs rather than worms cause the fibrosis related pathology the correlation between egg burden and fibrosis is weak (Kamden et al. 2018). We will be looking for genetic associations with worm burden and we have selected genes that have been or could be associated with either egg or worm burden and therefore a test that directly measures this burden is more appropriate than a proxy such as egg count. A candidate gene study of genetic associations with pathology would include a very different set of genes

 In the current study we also conduct at least one KK to check the correlation with both CAA and CCA tests. This is what is practical in the field for us. The CAA test is a true quantitative measure (van Dam et al., 2013), while the reflection from the CCA test has been modified for use as a quantitative test (Mewamba et al., 2021).

 Whilst we accept that multiple KK readings on consecutive days gives a more reproducible results than duplicate readings on a single day, we are planning to use the CCA as it is more reproducible from a single reading than the KK. 

 Kato Katz, the current standard method for detecting the egg burden requires 3 different readings from a non-homogenous stool sample. It also depends to a great extent the time of sampling. For these practical reasons, we will base worm burden quantification on CAA and reader assisted CCA.

**The authors propose to use the top 25% positive participants as participants with high burden. Considering the WHO classification for *S. mansoni* infection (light < 100; 100 < Moderate <400 or heavy > 400), what would be the contingency plan if participants in the top 25% have a burden lower than 400 EPG? Or 100EPG?**

 The egg burdens vary by community, where some communities will have predominantly light burdens or heavy burdens and these burdens will depend on the intensity of exposure and the history of MDA in the region. In so far as intensity of infection is controlled by genetic factors, we assume that a person with a worm burden in the top 20% in a community with low intensities of infection would be likely to be also in the top 20% in a community with heavy worm burdens and therefore the absolute intensity of infection is not relevant. We therefore use relative burdens, where we define the highest burden as those over the 80 ^th^ percentile of worm burdens. These are are refered to as the top 20% in the protocol. In the case control study, we compare the top 25% with the bottom 25%.  With this definition, we therefore do not use the WHO definitions of light, moderate and heavy egg burdens.

**The Figure 1 is quite confusing. The authors should consider revising it. Also, excluded samples should be mentioned. In addition, participants should be screened for clinical signs of anemia and excluded prior to blood collection.**

 We have revised this figure as shown in Figure1_flow_revised.docx

**The authors should consider screening for other confections such as malaria etc. - generally co-endemic in these regions, as co-infections could affect the susceptibility of Schistosomiasis infection.**

 This is an important consideration. We note other helminth infections when screening samples by the KK test but these have been rare so far (<5%). We are aware of evidence that schistosome infections can modify malaria infections and drug response but there seems to be less work on the effect of malaria in schistosomiasis. We will screen for malaria infections if possible and include this as a covariate in the analysis. In the case control study, we actually screened for fever, diarrhea and infections with other parasitic worms as described in section “Additional data recorded per participant”.

**Risk factors of susceptibility/Resistance to Schistosomiasis infection will be valuable information to have.**

 We have a questionnaire that seeks to capture environmental and demographic risk factors for schistosomiasis. In section “Data management” we stated that “Data will be collected in standardised forms as shown in supplementary data.”An example is the Uganda case control study case report form. Among these are included important variables like age, gender and sanitation facilities.

**It is not mentioned if the participants will be treated with Praziquantel at the end of the study.**

 We confirm that participants will be treated with Praziquantel. These studies are nested in the National Control Programme, where we sample immediately before treatment with Praziquantel. We stated in the section “Screening procedures” that  “All children will be treated with Praziquantel by the national Ministry of Health Mass Drug Administration teams according to the recommendation of the World Health Organization (World Health Organisation, 2006).”

**For the case-control study, the authors should correct for socio-demographic data to ensure minimal variation across the groups. For example, to fully attribute the difference in infection intensity to the genetics, the authors should make sure factors like age; gender; BMI etc. are not different between cases and controls. This will aid in minimizing confounding factors.**

 Yes, this is actually the analysis plan. We mention we control for these factors in section Study 2: Case control association study under subtopic  “Data analysis” we mention that “Age, sex, MUAC and BMI will also be used as additional covariates” in our analysis.

 MUAC is mid upper arm circumference

 BMI Body mass index

 REFERENCES:

 van Dam GJ, de Dood CJ, Lewis M, *et al.*: **A robust dry reagent lateral flow assay for diagnosis of active schistosomiasis by detection of *Schistosoma *circulating anodic antigen.** *Exp Parasitol. *2013; **135**(2): 274–282.

 van Dam GJ, de Dood CJ, Lewis M, Deelder AM, van Lieshout L, Tanke HJ, van Rooyen LH, Corstjens PL. A robust dry reagent lateral flow assay for diagnosis of active schistosomiasis by detection of Schistosoma circulating anodic antigen. Exp Parasitol. 2013 Oct;135(2):274-82. doi: 10.1016/j.exppara.2013.06.017. Epub 2013 Jul 10. PMID: 23850995; PMCID: PMC3797160.

 Kamdem, Severin Donald, Roger Moyou-Somo, Frank Brombacher, and Justin Komguep Nono. 2018. ‘Host Regulators of Liver Fibrosis During Human Schistosomiasis’. *Frontiers in Immunology* 9: 2781. https://doi.org/10.3389/fimmu.2018.02781.

 Mewamba EM, Tiofack AAZ, Kamdem CN, Ngassam RIK, Mbagnia MCT, Nyangiri O, Noyes H, Womeni HM, Njiokou F, Simo G. Field assessment in Cameroon of a reader of POC-CCA lateral flow strips for the quantification of Schistosoma mansoni circulating cathodic antigen in urine. PLoS Negl Trop Dis. 2021 Jul 14;15(7):e0009569. doi: 10.1371/journal.pntd.0009569. PMID: 34260610; PMCID: PMC8312929.

 World Health Organisation, ed. *Preventive Chemotherapy in Human Helminthiasis: Coordinated Use of Anthelminthic Drugs in Control Interventions ; a Manual for Health Professionals and Programme Managers*. Geneva: World Health Organization, 2006.
